# Atherosclerotic Renovascular Disease: A Re-evaluation of Lesion Severity and Intervention

**DOI:** 10.1016/j.xkme.2026.101406

**Published:** 2026-05-12

**Authors:** Arend JJ. Woittiez, Cornelis T. Postma, Peter W. de Leeuw

**Affiliations:** 1 Department of Internal Medicine, Hospital Group ZGT, Almelo/Hengelo, The Netherlands; 2 Department of Internal Medicine, Radboud University Medical Center, Nijmegen, The Netherlands; 3 Department of Internal Medicine, University Medical Centre, Maastricht, The Netherlands

**Keywords:** atherosclerotic renal artery stenosis, renovascular disease, hypertension, intervention, autoregulation, fractional flow reserve

## Abstract

Atherosclerotic renal artery stenosis (ARAS) remains a complex contributor to hypertension and kidney disease. Although randomized trials failed to show benefit of revascularization on top of medical therapy over medical therapy alone, mounting evidence suggests that earlier stages of ARAS may still be clinically relevant. In this review, we will reevaluate assumptions about the relationship between lesion severity and clinical outcomes and highlight the discrepancy between angiographic definitions of significant stenosis and functional renal impairment. We suggest that with all degrees of angiographically identified ARAS, particularly in patients with refractory hypertension, Kidney failure, or flash pulmonary edema, the hemodynamic importance of the stenosis should be estimated. In this regard, a pressure drop of 10% or more across the stenosis is an indication that hypertension may be related to the obstruction. Because the classical definition of a hemodynamically significant stenosis precludes earlier and potentially more adequate opportunities for intervention, it is presently unknown if patients with ARAS of less than 60% stenosis would benefit from mechanical treatment. We suggest reevaluating the thresholds for intervention and call for new trials focused on early-stage atherosclerotic renal vascular disease.

Atherosclerotic renal artery stenosis (ARAS) is closely linked to systemic atherosclerosis and associated with increased mortality.^1^ Nevertheless, despite its high prevalence in elderly patients and those with resistant hypertension, the clinical relevance of ARAS and the potential benefits of interventional treatment remain contentious. Guidelines on the treatment of hypertension mainly consider patients with primary hypertension but there is evidence that blood pressure targets should be lower in patients with renal artery stenosis.^2^

Although renal artery obstruction may be caused by a variety of abnormalities,^3^ ARAS is the most prevalent one and the one that gives rise to considerable discussion and controversies. There is general agreement that patients with ARAS should be vigorously treated with an optimal scheme of antihypertensive drugs, lipid lowering agents, and aspirin,^4^ but controversy exists regarding the need for restoring the patency of an obstructed renal artery. This is largely so because several trials failed to show any benefit of angioplasty, with or without stenting, over and above medical treatment alone.^5^^,^^6^ Accordingly, there is a tendency now among clinicians not to look actively for renal artery stenosis as it is deemed to be without specific therapeutic consequences.

Considering our current pathophysiological thinking about the effect of ARAS on blood pressure and renal function, it is surprising that intervention trials did not show what they were expected to show. However, negative trial results do not necessarily imply that the treatment under scrutiny should be abandoned altogether. It may still be that a specific subgroup of patients could benefit from restoration of the renal artery.^7^ Alternatively, our pathophysiological concepts could be wrong. Indeed, recent data cast doubt on the current paradigm of ARAS and call for a new and better-founded approach. The aim of the present minireview is to summarize these data and to provide a research agenda for the near future. We shall address 3 questions about the relation between ARAS and hypertension, about the so-called significance of the stenosis, and about the impact of interventions in patients with less severe stenosis.

### Question 1: What is the True Relationship Between ARAS and Hypertension?

The classical Goldblatt model, based on experimental renal artery occlusion in dogs, demonstrated that renal hypoperfusion results in a rise in blood pressure,^8^ likely via activation of the renin-angiotensin-aldosterone system. However, extrapolation to human ARAS is problematic. Like generalized atherosclerosis, ARAS is a slowly worsening disease, with an estimated progress rate of the stenosis of 10%-15% per year.^9^^,^^10^ Even though statin therapy can significantly slow down progression, it cannot stop it.^11^ Unfortunately, our knowledge of the natural history of the disease is still far from complete and, in particular, it is uncertain which patient is likely to progress toward more severe stenosis and which one will remain stable under optimal medical treatment.

During progression, compensatory mechanisms will be activated which mitigate the initial hemodynamic insult. Consequently, the prevalence of true renovascular hypertension, ie, hypertension directly related to renal artery narrowing, is lower than generally assumed. Indeed, most cases of renal artery stenosis in men have been present for many years before they are detected.^10^

The definite proof of a causal relationship between hypertension and ARAS is that relief of the stenosis cures or improves the hypertension. At the end of the 20th century several reports on surgical repair of the ARAS demonstrated improvement of blood pressure, but complete cure of hypertension was rare.^12^ Additionally, most of the reports were case histories or based on less well designed trials.^13^ Later on, randomized trials, which evaluated the effect of percutaneous transluminal renal angioplasty with or without stenting on top of medical treatment versus that of medical treatment alone did not show an appreciable advantage of dilatation.^5^ In contrast, observational data in patients with fibromuscular dysplasia or traumatic stenosis show that blood pressure can, indeed, normalize after renal angioplasty,^14^^,^^15^ suggesting that a causal relationship between flow obstruction in the renal artery and hypertension does exist. However, kidney function in patients with fibromuscular dysplasia is well preserved,^16^ whereas patients with ARAS often exhibit irreversible vascular remodeling and nephron loss, which may limit the impact of revascularization. Moreover, a substantial portion of patients with incidentally detected ARAS is normotensive, challenging the assumption that ARAS is the sole cause of hypertension.^17^ Thus, there must be additional factors other than the stenosis per se that cause elevation of blood pressure. Taken together, the available data suggest that in the category of elderly patients with advanced atherosclerosis and ARAS, the renovascular component of the hypertension—if any—is of minor importance.

### Question 2: What Does the Concept of Significant Renal Artery Stenosis Mean?

In a recent KDIGO document, the authors defined a significant stenosis as a (2-dimensional) luminal diameter reduction of the renal artery of at least 70%.^18^ This threshold has also been adopted in clinical guidelines and some trials as an indication for angioplasty. But what are the arguments for this threshold? In fact, this cutoff is highly arbitrary and poorly validated. In the renal artery, on average only 2% of the actual plaque volume encroaches on the lumen as reflected by angiographic narrowing of the lumen.^19^ Angiographically determined luminal narrowing may, therefore, be only the tip of the iceberg as almost all of the plaque volume is hidden beneath the luminal surface.^20^ In other words, by the time a visible luminal reduction of 70% is reached, autoregulatory mechanisms are exhausted, and there must be substantial intrarenal damage.^21^ Indeed, experimental studies indicate that even moderate stenoses (30%-60%) can already induce relevant changes in renal perfusion, oxygenation, and downstream inflammation.^22^, ^23^, ^24^ This means that when we try to restore the patency of a renal artery with 70% stenosis, improvement of kidney function and blood pressure is no longer feasible.^23^ The inevitable conclusion, therefore, is that we cannot know whether a stenosis is significant on the basis of angiographic information alone.

Regardless of the degree of a radiologically determined stenosis, an estimate of its hemodynamic significance should follow, particularly in patients with refractory hypertension, flash pulmonary edema, or decrease in kidney function.^25^ Functional measurements related to renal artery stenosis include translesional pressure gradients and fractional flow reserve after a hyperemic stimulus. A hallmark paper showed that a pressure drop over the stenosis of 10% or more proved to be an excellent criterion to define a hemodynamically relevant stenosis.^22^ However, data on the relationship between pressure gradients or other functional tests and severity of ARAS are still insufficient.^26^

Cardiologists have developed the concept of fractional flow reserve as a method to estimate the hemodynamic severity of a coronary artery stenosis.^27^ Although it is tempting to apply such measurements also as a proxy for the hemodynamic significance of a renal artery stenosis, this is not without problems. With ARAS gradually developing, the kidney is able to adapt to disturbances in renal blood flow and renal oxygen consumption by means of autoregulatory adaptations behind the stenosis. When autoregulation is maximally activated, a hyperemic stimulus will no longer lead to an increase in flow. However, when the kidney is irreparably damaged, there will be no flow response after the stimulus either. Thus, even with such hemodynamic measurements it will often be impossible to define the stage of the stenotic process. Blood oxygenation level-dependent magnetic resonance imaging (MRI) or, more recently, multiparametric MRI have emerged as innovative techniques to assess single kidney function reduction in patients with ARAS but their place in diagnosis still needs further confirmation.^28^^,^^29^

Biomarkers such as endothelin-1, C-reactive protein, N-GAL, and other cytokines as well as certain micro-RNAs (eg miR-126 and miR-210) are often elevated in patients with ARAS and hold promise for the future as an adjunct to assess the degree of inflammation and vascular damage in the kidney behind the stenosis.^30^ However, these markers are not very specific for ARAS, and many of these assays are not yet ready for daily clinical practice.

If we cannot already establish the hemodynamic significance of a renal artery stenosis, we certainly cannot its clinical significance in terms of expected improvements in blood pressure or glomerular filtration rate after mechanical treatment. This is further illustrated by the differences in inclusion criteria of the major intervention trials. For instance, in the DRASTIC, the EMMA, the Scottish and Newcastle Renal Artery Stenosis Collaborative Group trial, and in the STAR trial, a stenosis severity of 50% or more was required for inclusion.^31^, ^32^, ^33^, ^34^ The NITER-and the RASCAD trials, on the contrary, used a criterion of 70% or more.^35^^,^^36^ In the CORAL trial, the degree of stenosis even had to be at least 80% or at least 60% with a pressure gradient of at least 20 mm Hg.^37^ In the ASTRAL study, it was not only the degree of stenosis, but the uncertainty of the clinician about the benefit of revascularization that drove inclusion into the study.^38^ The RADAR trials did not specify the degree of stenosis but used duplex criteria for inclusion.^39^ All this is an illustration of the fact that we do not know and have no consensus about what a significant—or even a moderate or mild—stenosis is.

### Question 3: Is There any Evidence to Consider Lower Degrees of ARAS for Intervention?

ARAS results in progressive loss of renal mass and function, and a substantial proportion of patients will develop chronic renal failure.^40^^,^^41^ The rate of complications seems to increase already with low-grade (<50%) stenosis.^42^^,^^43^ Thus, although low-grade stenoses are usually considered to be of no hemodynamic significance, they may very well be clinically relevant. This aligns with the correlation that we found between the degree of ARAS and the estimated glomerular filtration rate on the one hand and the prevalence of hypertension on the other.^44^ Although this does not necessarily mean that patients with low-grade stenosis would benefit from earlier interventions, the data provide at least some support for the proposition to set up a study to assess the effect of earlier intervention on top of intensive medical therapy.^45^ If flow alterations and local inflammation already occur early as a result of the local atherosclerotic process, intervention by means of a dilation procedure at this stage may be considered as well to prevent irreversible renal injury. The latter has to be weighted, of course, against procedural complications in patients who may have remained stable otherwise.^46^

### Clinical Implications

From the foregoing, we can conclude that that the relationship between ARAS and hypertension is far more complex and goes further than an impediment of flow alone. It is not clear what we should consider a significant stenosis as seen on computed tomography, MRI, or intra-arterial angiography. At any rate, complications, both locally and systemically, occur early in the development of ARAS. We speculate, therefore, that in patients with low-grade ARAS, an intervention on top of medical treatment may halt the progression of the atherosclerotic process and thereby prevent further renal damage (and augmented systemic atherosclerosis). We acknowledge that, at present, there is no direct proof for this approach and that this should be tested in a prospective trial.

In patients with clinical signs of so-called renovascular disease (flash pulmonary edema, treatment-resistant hypertension, and kidney failure) screening for ARAS may be performed by means of computed tomography angiography or magnetic resonance angiography. To date, we are not aware of any studies that have utilized biochemical markers for detecting hemodynamically relevant ARAS. However, also in younger patients, who do not suffer from advanced atherosclerosis, screening should be considered. Regardless of the degree of the stenosis on imaging, only functional tests such as pressure gradients, fractional flow reserve measurements and (split) renal function under renin-angiotensin-aldosterone system inhibition, and multiparametric MRI could be helpful in the decision to proceed to intervention. Although this still requires an invasive procedure, there seems to be no role anymore for intraarterial renal angiography unless a computed tomography angiography or magnetic resonance angiography is inconclusive.
